# Performance of Thirteen Clinical Rules to Distinguish Bacterial and Presumed Viral Meningitis in Vietnamese Children

**DOI:** 10.1371/journal.pone.0050341

**Published:** 2012-11-28

**Authors:** Nguyen Tien Huy, Nguyen Thanh Hong Thao, Nguyen Anh Tuan, Nguyen Tuan Khiem, Christopher C. Moore, Doan Thi Ngoc Diep, Kenji Hirayama

**Affiliations:** 1 Department of Immunogenetics, Institute of Tropical Medicine (NEKKEN), Nagasaki University, Nagasaki City, Japan; 2 Department of Pediatrics, University of Medicine and Pharmacy at Ho Chi Minh City, Ho Chi Minh City, Vietnam; 3 Children's Hospital No.1, Ho Chi Minh City, Vietnam; 4 Department of Pediatrics, Pham Ngoc Thach University of Medicine, Ho Chi Minh City, Vietnam; 5 Division of Infectious Diseases and International Health, Department of Medicine, University of Virginia, Charlottesville, Virginia, United States of America; 6 Global COE Program, Nagasaki University, Nagasaki City, Japan; University of Medicine & Dentistry of New Jersey – New Jersey Medical School, United States of America

## Abstract

**Background and Purpose:**

Successful outcomes from bacterial meningitis require rapid antibiotic treatment; however, unnecessary treatment of viral meningitis may lead to increased toxicities and expense. Thus, improved diagnostics are required to maximize treatment and minimize side effects and cost. Thirteen clinical decision rules have been reported to identify bacterial from viral meningitis. However, few rules have been tested and compared in a single study, while several rules are yet to be tested by independent researchers or in pediatric populations. Thus, simultaneous test and comparison of these rules are required to enable clinicians to select an optimal diagnostic rule for bacterial meningitis in settings and populations similar to ours.

**Methods:**

A retrospective cross-sectional study was conducted at the Infectious Department of Pediatric Hospital Number 1, Ho Chi Minh City, Vietnam. The performance of the clinical rules was evaluated by area under a receiver operating characteristic curve (ROC-AUC) using the method of DeLong and McNemar test for specificity comparison.

**Results:**

Our study included 129 patients, of whom 80 had bacterial meningitis and 49 had presumed viral meningitis. Spanos's rule had the highest AUC at 0.938 but was not significantly greater than other rules. No rule provided 100% sensitivity with a specificity higher than 50%. Based on our calculation of theoretical sensitivity and specificity, we suggest that a perfect rule requires at least four independent variables that posses both sensitivity and specificity higher than 85–90%.

**Conclusions:**

No clinical decision rules provided an acceptable specificity (>50%) with 100% sensitivity when applying our data set in children. More studies in Vietnam and developing countries are required to develop and/or validate clinical rules and more very good biomarkers are required to develop such a perfect rule.

## Introduction

Accurate and rapid diagnosis of acute bacterial meningitis (ABM) is essential as successful disease outcome is dependent on immediate initiation of appropriate antibiotic therapy [1], [2]. Differentiating ABM from presumed acute viral meningitis (pAVM) often proves challenging for clinicians as their symptoms and laboratory tests are often similar and overlapping. Classical clinical manifestations of ABM in infants and children are usually difficult to recognize given the absence of meningeal irritation signs and delayed elevation of intracranial pressure. In addition, the various parameters examined in the cerebral spinal fluid (CSF) are less discriminative in children than in adults, especially in enterovirus meningitis where the CSF parameters may be similar to bacterial meningitis values. The vast majority of patients with acute meningitis are administered broad-spectral antibiotics targeting ABM while awaiting results of definitive CSF bacterial cultures. In the absence of ABM, this practice may enhance the local frequency of antibiotic resistance [3], cause adverse antibiotic effects [4], and high medical costs [5]. Thus, it is not only important to recognize ABM patients who promptly require antimicrobial therapy, but also pAVM patients who do not need antibiotics or hospital admission at all. An ideal diagnostic rule should demonstrate 100% sensitivity in detecting bacterial meningitis [6], while retaining a high specificity.

Unfortunately, no single clinical symptom or laboratory test has differentiated ABM from pAVM with 100% sensitivity and high specificity [7], [8]. More recently, numerous researchers have investigated potential clinical decision rules that recognize ABM from pAVM including: Thome [9], Spanos [10], Hoen [11] (also called Jaeger et al [12]), Freedman [13], Nigrovic [14], Oostenbrink [15], Bonsu 2004 [16], Brivet [17], Schmidt [18], De Cauwer [19], Chavanet [20], Dubos [21], Bonsu 2008[22], Tokuda [23], and Lussiana [24]. A few rules have included complicated multivariate models that require the use of a computer [10], [11], while others have used scoring systems [9], [15], tree model decisions [23], or a simple list of items [13], [14], [17], [18], [19], [20], [21], [22]. These clinical decision rules require extensive test prior to their use in hospitals [25] and have rarely been compared in a single study. In addition, several rules are yet to be tested by independent researchers [17], [21], [22], [23], [24] or tested in children [17], [23]. The Nigrovic's rule, also called Bacterial Meningitis Score (BMS) [14], performed perfectly in several studies [8], [26], [27], [28], [29], [30], but failed to provide 100% sensitivity in other independent data sets [7], [19], [20], [31]. Simultaneous test and comparison of these rules is required to enable clinicians to select an optimal rule to limit the number of patients being unnecessarily treated with antibiotics, and to guarantee that patients with bacterial meningitis receive appropriate antibiotics.

## Materials and Methods

### Identification of clinical rules

Two electronic databases including PubMed and Scopus were searched for suitable clinical rules. The search terms used were as follows: “dengue AND (rule OR score)”. We supplemented these searches with a manual search of articles that developed and/or compared clinical rules. Since we aimed to find the clinical rule that could be applied in our hospital and test the generalizability of clinical rules [32], no restrictions were applied with respect to country, year, and language of studies that developed clinical rules. A total of 15 clinical rules were identified. Among them the Bonsu 2008 [22] and Dubos rules [21] were not tested as band leukocytes and procalcitonin were not available in our hospital.

### Study design

The current study was performed at the Infectious Department of Pediatric Hospital Number 1, Ho Chi Minh City, Vietnam. The hospital is a tertiary pediatric hospital in southern Vietnam with 1200 beds. It was a retrospective cross-sectional analysis of the clinical signs and laboratory tests obtained from previously healthy children (≤15 years) that were diagnosed with acute meningitis. Discharge diagnosis was reviewed to identify meningitis patients based on the International Classification of Diseases, 10^th^ Revision (ICD-10) with the following codes: G00, G00.x, G01*, G02.0*, G03, and G03.x. The study was approved in advance by the Ethical Review Committee of the Pediatric Hospital Number 1, Ho Chi Minh City, Vietnam. Written informed consent from the patients or their parents was waived by the Committee, because all data were retrospectively collected after the discharge of patients and numerically coded to ensure patient anonymity.

The entry criteria were as follows: children with proven acute bacterial meningitis (ABM) or presumed acute viral meningitis (PAVM), who had received a lumbar puncture between December 2003 and December 2008. Patients exhibiting blood-contaminated CSF (CSF erythrocyte count >10,000 cells/μL) [33], tuberculous meningitis, HIV infection, immune depression, and those found to have histories of pulmonary tuberculosis, liver diseases such as autoimmune disease, alcoholic liver disease and metabolic disease, kidney disease, neurosurgical disease or had undergone recent neurosurgery were excluded from the study. Neonates (less than 28 days old) and patients with missing laboratory variables listed in Table 1 were also excluded.

**Table 1:** Characteristic of variables used in the clinical decision rules to distinguish ABM from pAVM.

Variables	Scores using equation			List of items					Classified scores				Tree model
	Spanos (1989)	Hoen (1995)	Bonsu (2004)	Freedman (2001)	Nigrovic (2002)	Brivet (2005)	Schmidt (2006)	De Cauwer (2007)	Thome (1980)	Oostenbrink (2004)	Chavanet (2007)	Lussiana (2011)	Tokuda (2009)
**Clinical variables**													
Age	⊕		⊕	⊕									
Admission month	⊕												
Symtoms duration										⊕			
Seizure					⊕	⊕			⊕				
Vomit										⊕			
Body temperature									⊕				
Disturbed consciousness						⊕			⊕	⊕			⊕
Focal neurological						⊕							
Shock						⊕							
Meningeal irritation										⊕			
Cyanosis										⊕			
Purpura or petechiae									⊕	⊕			
**Blood variables**													
WBC		⊕							⊕				
Neutrophils %													
Neutrophil count				⊕	⊕								
Neutrophil band count													
Glucose		⊕											
CRP								⊕		⊕			
**CSF variables**													
Gram stain	⊕			⊕	⊕								⊕
WBC				⊕			⊕		⊕		⊕	⊕	
Neutrophils %								⊕	⊕		⊕		⊕
Neutrophil count	⊕	⊕	⊕		⊕								⊕
Protein		⊕	⊕	⊕	⊕		⊕	⊕	⊕		⊕	⊕	
Glucose				⊕				⊕	⊕		⊕	⊕	
CSF/blood glucose ratio	⊕			⊕									
Lactate							⊕						
Threshold	pABM* ≥0.1			≥1 item					Complex judge				

* Probability of ABM (pABM).

Proven ABM was diagnosed if the patient demonstrated CSF pleocytosis (CSF leukocyte count >7 cells/μL) [34], [35] in addition to one of the following test results: (1) positive CSF culture for bacterial pathogens, (2) positive CSF latex agglutination test, or (3) positive blood culture. PAVM was defined as patients with a pleocytosis in the CSF (CSF leukocyte count >7 cells/μL) in addition to positive culture for viral pathogens or rapid remission without extensive antibiotic therapy combined with an absence of any four criteria of proven ABM [10], [14], [20], [26], [36], [37].

Blood cultures were performed using 5% sheep blood agar before 2005 and a BACTEC 9240 system instrument (BD Biosciences, China) from 2005. CSF culture was done on 7% horse blood agar and 5% chocolate blood agar plates and incubated at 36°C for 24 h. Observed colonies were further identified by standard microbiological methods. Viral culture was not routinely performed, only five CSF samples were sent to Pasteur Institute (Ho Chi Minh City, Vietnam) for virus isolation.

At the time of admission, the relevant patient history regarding clinical symptoms and signs, and laboratory parameters listed in the Table 1 was collected. Clinical signs and symptoms that were not noted in the patient medical record were coded as normal.

### Data analysis

All information was entered into a Microsoft Office Excel 2007 computerized database. Missing clinical signs and symptoms were not included and the number of patients per group was also adjusted before analysis. Our analysis showed that there were no significant differences in selected variables between patients with and without missing data.” into the data analysis (page 6).

A score, judge, or probability of ABM (pABM) was calculated from each patient for each of the clinical decision rules according to the authors of the rules (Method S1). The overall accuracy of these rules represented by area under a receiver operating characteristic curve (ROC-AUC) was compared by the method of DeLong [38] using MedCalc statistical software (11.0, MedCalc Software bvba, Belgium). AUC values ≥0.5, 0.75, 0.93, or 0.97 were considered as fair, good, very good, or excellent accuracy [39]. The sensitivity and specificity of each rule was then calculated using our patient data set. To do so, we applied the thresholds indicated by the authors of the rules and by our own ROC analyses. The rules demonstrating 100% sensitivity were further analyzed to compare their specificity using the McNemar test [8].

The minimal required sample size and power of comparison were calculated using the MedCalc statistical software based on 5% type I error rate and 20% type II error rate. Assuming that ROC-AUCs of all clinical rules are at least 90% compared to the null hypothesis value 70% [22] , the required sample size was 48 subjects per group in this case.

**Figure 1:**
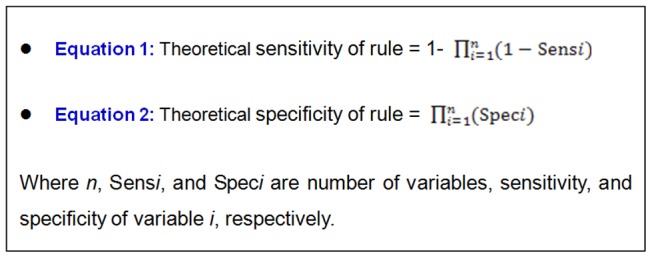
Equation for calculation of theoretical sensitivity and specificity of simple list of items rule with cut-off value at one item.

**Figure 2:**
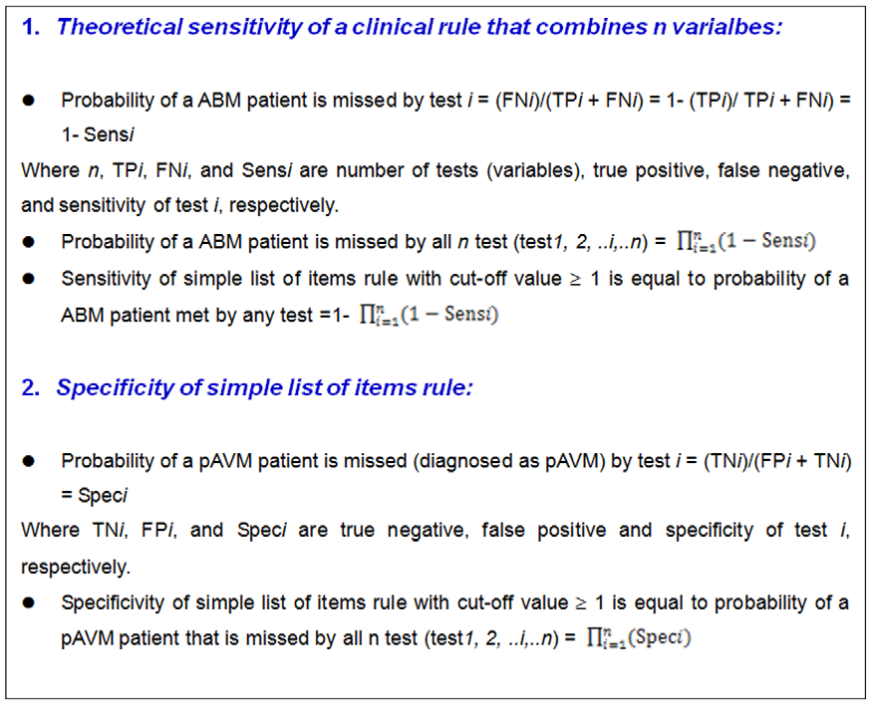
Explanation for calculation of theoretical sensitivity and specificity. The theoretical sensitivity is the likelihood of sensitivity of the clinical rule after combining *n* tests, thus its values is depend on the individual sensitivity of each test. For example, a clinical rule combining two tests with sensitivities at 90% and 80%, respectively, the likelihood of the combined sensitivity (of the clinical rule of two tests) is calculated as 1–(1–0.90)×(1–0.80) = 0.98 or 98%. Therefore, combination of several tests will enhance the rule's sensitivity. In contrast, a clinical rule combining two tests with specificities at 80% and 70%, the likelihood of the combined specificity (of the clinical rule of two tests) will be decreased as the follow calculation: 0.80×0.70 = 0.56 or 56%.

In order to explain the limitation of Nigrovic's rule, we calculated the theoretical sensitivity and specificity of simple list of items rule with cut-off value at one item. Since selected variable demonstrated an independent predictor of ABM [14], the theoretical sensitivities and specificities of the simple list of items rule with cut-off value ≥1 can be derived from individual sensitivity and specificity of each variable as presented by equation 1 and 2, respectively (Figure 1). The individual sensitivity and specificity of each variable were derived from the current study unless otherwise stated. The method calculation was described in the (Figure 2).

## Results

### Characteristic of patient population

Between December 2003 and December 2008, 192 patients met our inclusion criteria. A total of 63 patients were excluded from the final analysis due to the following reasons: (1) age of 0–28 days (n = 34), (2) traumatic lumbar puncture (n = 14), (3) recent neurosurgery or head injury (n = 12), or (4) HIV infection (n = 3). The high number of excluded patients could be explained by the characteristics of the tertiary hospital. A total of 129 patients including 80 ABM (62%) and 49 PAVM (38%) patients were selected for the final analysis (Table 2). Among the 80 patients with proven ABM, death occurred in 6.3% (n = 5), and neurological sequelae was observed in 25% (n = 15, Table 2). Of the 80 ABM cases, bacterial pathogen was identified in the CSF Gram-stain of 34 cases (43%), in the CSF culture of 39 cases (49%), blood culture of 18 patients (23%), in the blood culture alone of one patient (1.2%), and by latex agglutination in 65 patients (81%). Bacterial infections were caused by *Haemophilus influenzae* (n = 49, 61.3%), *Streptococcus pneumoniae* (n = 26, 32.5%), *Streptococcus agalactiae* (n = 1, 1.3%), *Neisseria meningitides* (n = 1, 1.3%), *Escherichia coli* (n = 2, 2.5%) and *Morganella morganii* (n = 1, 1.3%). Of the 49 PAVM cases, Herpes simplex virus 1 was the only viral pathogen isolated (n = 2).

**Table 2:** Characteristics of the 129 patients in this study.

Characteristic	ABM n (%) or mean ± SD	pAVM n (%) or mean ± SD
**Number of patients**	80	49
**Age ≤ 12 month**	49 (62)	13 (28)
**> 12 month**	31 (38)	36 (72)
**Sex: male**	50 (63)	33 (67)
**Duration of illness (days, median, 95% CI for the median)**	3 (3–5)	2 (2–3)
**Hospitalization days**	16.3±8.8	5.3±2.6
**Nausea**	3 (4)	4 (8)
**Vomiting**	47 (60)	31 (62)
**Fever**	78 (98)	48 (96)
**Purpura**	0 (0)	0 (0)
**Cyanosis**	7 (9)	1 (2)
**Seizure**	54 (68)	11(22)
**Fever**	59 (74)	36 (72)
**Cyanosis**	13 (16)	1 (2)
**Purpura or petechiae**	4(5)	0 (0)
**Meningeal signs**	28 (35)	20 (40)
Bulging **fontanelle**	37 (46)	11 (22)
**Altered mental status**	35 (44)	4 (8)
**Focal neurological deficits**	23 (29)	5 (10)
**Shock**	3 (4)	0 (0)
**Blood WBC**	15,398±9,033	13.420±4,989
**Blood neutrophil %**	58.5±18.1	53.0±21.4
**Blood neutrophil count**	9,776±8,224	7.298±4,709
**Blood glucose (mg%)**	84.5±30.4	89.8±18.2
**Blood CRP**	136.7±97.5	25.0±47.9
**CSF WBC**	2,946±5,809	136±215
**CSF neutrophils %**	71±21	36±23
**CSF neutrophil count**	2,469±4,920	36±48
**CSF protein (g/L)**	1.13±0.70	0.39±0.31
**CSF glucose (mg%)**	26.1±19.6	56.9±12.9
**CSF/blood glucose ratio**	0.34±0.26	0.65±0.19
**CSF lactate (mmol/L)**	7.0±4.3	2.1±0.7
**Blood culture (+)**	18 (23)	
**Gram-stain (+)**	34 (43)	
**CSF culture (+)**	39 (49)	
**Latex (+)**	65 (82)	
**Death**	5 (6)	0 (0)
**Neurological sequelae or death**	20 (25)	0 (0)

### Comparison of clinical rules

**Figure 3:**
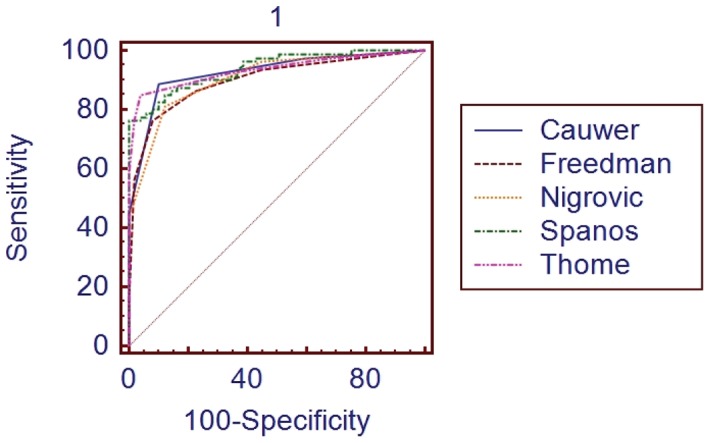
ROC curves of five best clinical rules for differential diagnosis of ABM from PAVM. The AUCs of ROC curves were 0.927 for De Cauwer rule, 0.900 for Freedman, 0.907 for Nigrovic, 0.938 for Spanos, and 0.935 for Thome. Pairwise comparison of all ROC-AUCs showed no significant difference of the five selected rules.

The overall accuracy of the rules was explored by calculation of the ROC-AUCs. All 13 clinical rules possessed AUC values between 0.75 and 0.94, indicating good accuracy (Table 3 and Figure S1) [39]. The Spanos rule had the highest AUC at 0.938. However, when comparing with the other four best rules (De Cauwer, Freedman, Nigrovic, and Thome), the Spanos rule was not significantly better by Delong method [38] (P>0.05, Figure 3).

**Table 3:** Accuracy comparison of clinical rules.

Rule	AUC	Cut-off values	Sensitivity % (95% CI)	Number of ABM patients missed by the rule	Specificity % (95% CI)
**Thome**	0.935	≥2#	92.5 (84.4–97.2)	6	65.3 (51.2–78.8)
**Spanos**	0.938	**pABM>0.04** *	**100 (96.3–100)**	**0**	**^a^24 (13.1–38.2)**
		pABM≥0.10#	98.7 (93.2–99.9)	1	34 (21.2–48.8)
**Hoen**	0.883	pABM>0.0026	**100 (96.3–100)**	0	^a^4 (0.5–13.7)
		pABM≥0.10#	77.2 (65.4–85.1)	19	80 (67.7–89.2)
**Freedman**	0.900	≥1#	98.7 (93.2–99.9)	1	12.2 (5.8–26.7)
**Nigrovic**	0.907	≥1#	96.3 (91.2–98.7)	3	55.1 (46.9–59.0)
**Oostenbrink**	0.758	≥2⊕	100 (96.3–100)	0	^a^8 (2.2–19.2)
		≥8.5#	78.5 (66.8–86.1)	18	50 (35.5–64.5)
**Bonsu**	0.812	**pABM≥0.014**	**100 (96.3–100)**	0	**^a^8 (2.2–19.2)**
		pABM≥0.10#	92.4 (82.8–96.4)	7	28 (16.2–42.5)
**Brivet**	0.790	≥1#	81.3(71.0–89.1)	15	70 (55.4–82.1)
**Schmidt**	0.880	≥2#	58.8 (47.2–69.7)	33	100 (92.9–100)
**De Cauwer**	0.927	≥1#	98.7 (93.2–99.9)	1	40.8 (33.3–43.7)
**Chavanet**	0.878	≥2#	78.5 (66.8–86.1)	18	96 (86.3–99.5)
**Tokuda**	0.876	High risk	87.5 (78.2–93.8)	10	88 (75.7–95.5)
**Lussiana**	0.868	High risk	90.0 (81.2–95.6)	8	75.5 (61.1–86.7)

# Thresholds indicated by the authors of the rules.

⊕ Thresholds computed by ROC analysis to achieve 100% sensitivity.

* Probability of ABM (pABM).

Numbers in boldface indicate rule with 100% sensitivity.

When applying the thresholds indicated by the authors of the rules, no rule demonstrated 100% sensitivity, as prediction rules failed to identify six ABM patients by Thome, one ABM patient by Spanos, 19 ABM patients by Hoen, one ABM patients by Freedman, three ABM patients by Nigrovic, 18 ABM patients by Oostenbrink, seven ABM patients by Bonsu, 15 by Brivet, 33 ABM patients by Schmidt, one ABM patient by De Cauwer, 18 ABM patients by Chavanet, ten by Tokuda, and eight by Lussiana's rule. When applying the thresholds computed by our ROC analysis to achieve 100% sensitivity, all rules showed low specificity (< 25%). The Spanos's rule demonstrated the highest specificity at 24%, followed by Oostenbrink (8%), Bonsu (8%), Hoen's rules (4%), while the Freedman, Nigrovic, Thome, Brivet, Schmidt, De Cauwer, Chavanet, Tokuda, and Lussiana's rules could not achieve 100% sensitivity.

Our calculation showed that the theoretical sensitivity of Nigrovic's rule was 96.6% when computing the variables' sensitivity values observed in our study. The strength of the theoretical sensitivities was in the following order: Freedman  =  De Cauwer > Nigrovic > Schmidt > Brivet > Chavanet, which was almost identical to the order of real sensitivities performed in our data set (Table S1). The theoretical sensitivity of Nigrovic's rule was just slightly increased (98.1%) upon computing the variables' sensitivity values observed in Nigrovic's studies [14], further supporting that the rule is not perfect. Similarly, the strength of theoretical specificities was in the following order: Chavanet > Schmidt > Brivet > Nigrovic > De Cauwer > Freedman. These findings were similar to the order of real specificities in the data set. Furthermore, the correlation between the theoretical and real accuracy was analyzed by a Spearman rank test. Our results demonstrated that the theoretical sensitivity and specificity were highly correlated with real sensitivity and specificity, respectively (Figure S2). Overall, there was no statistical difference between theoretical calculations and real values in data sets in regards to sensitivity and specificity, suggesting that our calculation was correct.

## Discussion

To our knowledge, this is the first study that simultaneously tested more than ten prediction rules for clinical practice in meningitis. No clinical rule had superior overall accuracy compared to other rules. In addition, no rule provided 100% sensitivity with acceptable specificity (>50%). The overall accuracy of the two earliest rules (Spanos and Thomas rules) was not outperformed by recent developed rules, probably due to the similar epidemiology to the pre-vaccination era [10]. The high frequency of *H. influenzae* in our study could be explained by the lack of conjugate Hib vaccine in the Vietnamese national vaccination policy, and only a small number of children (0.5%) reportedly received conjugate Hib vaccine [40].

Among reported clinical decision models, the Nigrovic's rule [14] is the only rule that has been tested by more than three independent groups, and performed perfectly in several studies [8], [26], [27], [28], [29], [30]. However, it only provided 96.3% sensitivity in our study, which is also in the range of other independent data sets [7], [19], [20], [31] and well agreed with the theoretical sensitivity (96.64%) and specificity values (53.35%), explaining that the Nigrovic's rule could not identify all ABM patients in several data sets.

Based on these evidences and our equations, an ideal simple list of items clinical rule with theoretical sensitivity >99.99% and theoretical specificity >50% should include at least four independent variables that posses both sensitivity and specificity >85–90%. In addition, to improve the rule sensitivity without significantly reducing its specificity, we recommend adding additional variables with extremely high specificity (approximately 100%). We are not aware of more than three such conventional parameters to derive such an ideal rule. However, recent studies have proposed that blood procalcitonin [21], [41], CSF lactate [42], [43], [44], and blood C-reactive protein (CRP) [45] are very good biomarkers for bacterial meningitis. Upon addition of procalcitonin test (99% sensitivity and 83% specificity [37]), the theoretical sensitivity of Nigrovic's rule would be significantly increased from 96.64% to 99.77% (Calculation: 1−(1−0.9664)×(1−0.99) = 0.9997), while the theoretical specificity value would be dropped from 53.35% to 44.28% (Calculation: 0.5335×0.83 = 0.4428). However, these three parameters have rarely been measured in the same study and their usefulness and independent contribution in the differential diagnosis of ABM from pAVM are rarely evaluated [46], [47]. Thus, further studies are required to evaluate the contribution of these variables in the performance of clinical rules.

There were several limitations in our study. The first limitation was that the design was retrospective. Secondly, we only analyzed data from only one hospital. Therefore our results would be different from other hospitals, particularly in high-resources countries, where the epidemiology, clinical characteristics and outcome are different. Thirdly, our study focused on hospitalized patients in a big city. Therefore, further studies recruiting patients in clinics or local hospitals are required to further test these clinical rules. Fourthly, we could not confirm all pAVM patients as aseptic meningitis due to limited diagnosis in our hospital, which may affect the result. Another limitation is that the number of pAVM patients was much smaller than that of ABM, because several patients with extensive antibiotic therapy were excluded from criteria of pAVM. Finally, we were unable to include band leukocytes and blood procalcitonin, thus we could not test two promising Bonsu 2008 [22] and Dubos's [21] rules in the current study.

In conclusion, accurate bacterial meningitis is serious and the outcome is dependent on immediate initiation of appropriate antibiotic therapy. The best method for differentiating accurate bacterial meningitis from viral meningitis remains unclear. Several clinical decision rules have been derived to assist clinicians to distinguish between bacterial meningitis and viral meningitis, but barely tested and compared by independent studies. When applying our data set, no clinical rule provided an acceptable specificity (>50%) with 100% sensitivity. More studies in developing countries are required to confirm due to several limitations related to population and more accurate biomarkers are required to develop such a perfect rule.

## Supporting Information

Figure S1 **ROC curves of 13 clinical rules for differential diagnosis of ABM from PAVM when applying our data set.** The AUCs of ROC curves were 0.812 for Bonsu 2004, 0.790 for Brivet, 0.927 for De Cauwer, 0.878 for Chavanet, 0.900 for Freedman (upper panel), 0.883 for Hoen, 0.868 for Lussiana, 0.907 for Nigrovic, 0.758 for Oostenbrink (middle panel), 0.880 for Schmidt, 0.938 for Spanos, 0.935 for Thome, and 0.876 for Tokuda rule (lower panel). Pairwise comparison of all ROC-AUCs was shown as the follow: -Spanos rule was significantly better than Schmidt, Chavanet, Tokuda, Lussiana, Bonsu, Brivet, and Oostenbrink rule. -Thome rule was significantly better than Hoen, Chavanet, Tokuda, Lussiana, Bonsu, Brivet, and Oostenbrink rule. -De Cauwer rule was significantly better than Bonsu 2004, Brivet, and Oostenbrink rule. -Nigrovic rule was significantly better than Brivet and Oostenbrink rule. -Freedman rule was significantly better than Bonsu 2004, Brivet, and Oostenbrink rule. -Hoen rule was significantly better than Bonsu 2004, Brivet, and Oostenbrink rule. -Schmidt rule was significantly better than Bonsu 2004, Brivet, and Oostenbrink rule. -Chavanet rule was significantly better than Brivet and Oostenbrink rule. -Tokuda rule was significantly better than Bonsu 2004, Brivet, and Oostenbrink rule. -Lussiana rule was significantly better than Oostenbrink rule. -Other pairwise comparison showed no significant difference.
(TIF)

Figure S2 **Correlation between real and theoretical accuracy of six simple list of items rules.** The Spearman correlation showed an r value of 0.971, P = 0.001, n = 6 for sensitivity correlation, and r value of 1.0, P<0.001, n = 6.
(TIF)

Method S1 **Description and calculation of clinical rules.** The rules were derived from original studies.
(DOC)

Table S1 **Theoretical sensitivities and specificities of simple list of items rule calculated and compared using our data set.**
(DOC)
