# Supplementary figures and images for: Performance of Thirteen Clinical Rules to Distinguish Bacterial and Presumed Viral Meningitis in Vietnamese Children
Source: PLoS One. 2012 Nov 28;7(11):e50341. doi: 10.1371/journal.pone.0050341 (PMC3508924; doi:10.1371/journal.pone.0050341)

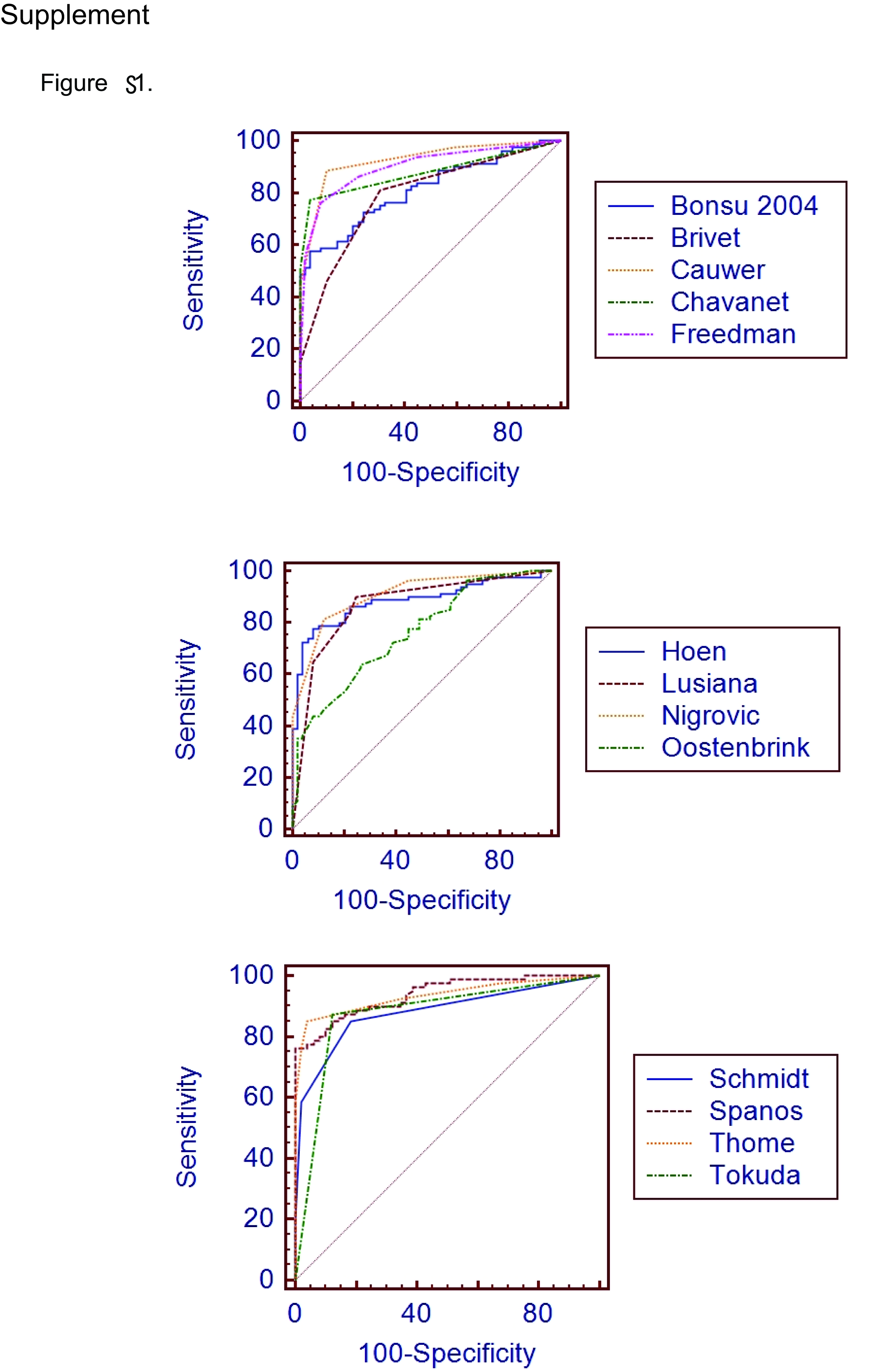

Supplement: Figure S1 — ROC curves of 13 clinical rules for differential diagnosis of ABM from PAVM when applying our data set. The AUCs of ROC curves were 0.812 for Bonsu 2004, 0.790 for Brivet, 0.927 for De Cauwer, 0.878 for Chavanet, 0.900 for Freedman (upper panel), 0.883 for Hoen, 0.868 for Lussiana, 0.907 for Nigrovic, 0.758 for Oostenbrink (middle panel), 0.880 for Schmidt, 0.938 for Spanos, 0.935 for Thome, and 0.876 for Tokuda rule (lower panel). Pairwise comparison of all ROC-AUCs was shown as the follow: -Spanos rule was significantly better than Schmidt, Chavanet, Tokuda, Lussiana, Bonsu, Brivet, and Oostenbrink rule. -Thome rule was significantly better than Hoen, Chavanet, Tokuda, Lussiana, Bonsu, Brivet, and Oostenbrink rule. -De Cauwer rule was significantly better than Bonsu 2004, Brivet, and Oostenbrink rule. -Nigrovic rule was significantly better than Brivet and Oostenbrink rule. -Freedman rule was significantly better than Bonsu 2004, Brivet, and Oostenbrink rule. -Hoen rule was significantly better than Bonsu 2004, Brivet, and Oostenbrink rule. -Schmidt rule was significantly better than Bonsu 2004, Brivet, and Oostenbrink rule. -Chavanet rule was significantly better than Brivet and Oostenbrink rule. -Tokuda rule was significantly better than Bonsu 2004, Brivet, and Oostenbrink rule. -Lussiana rule was significantly better than Oostenbrink rule. -Other pairwise comparison showed no significant difference. (TIF) [file pone.0050341.s001.tif]

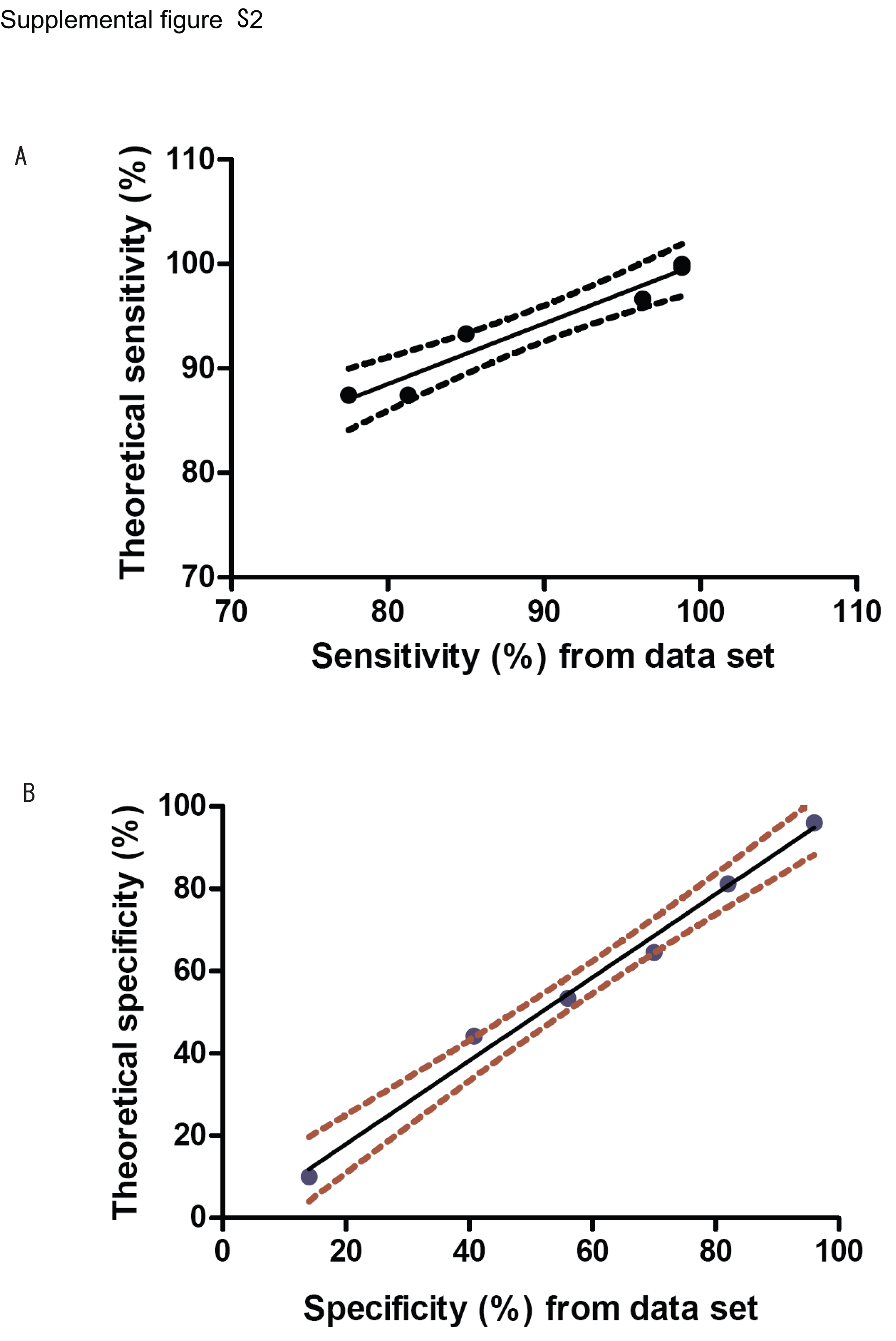

Supplement: Figure S2 — Correlation between real and theoretical accuracy of six simple list of items rules. The Spearman correlation showed an r value of 0.971, P = 0.001, n = 6 for sensitivity correlation, and r value of 1.0, P<0.001, n = 6. (TIF) [file pone.0050341.s002.tif]
