# Supplementary material for: Performance of Thirteen Clinical Rules to Distinguish Bacterial and Presumed Viral Meningitis in Vietnamese Children
Source: PLoS One. 2012 Nov 28;7(11):e50341. doi: 10.1371/journal.pone.0050341 (PMC3508924; doi:10.1371/journal.pone.0050341)
Supplement: Method S1 — Description and calculation of clinical rules. The rules were derived from original studies. (DOC) [file pone.0050341.s003.doc]

**Method S1: Description and calculation of clinical rules**

**Thome 1980 rule [1]**: used a table of classified score with cut-off value ≥ 2

| *Parameters* | *Points* | | |
| --- | --- | --- | --- |
|  | 0 | 1 | 2 |
| Body temperature | < 39.5°C | ≥ 39.5°C |  |
| Purpura | Absent |  | Present |
| Neurological signs | Absent | Present |  |
| CSF protein (g/L) | < 0.9 | 0.9 -1.4 | ≥ 1.4 |
| CSF glucose (mg%) | > 35 | 35-20 | ≤ 20 |
| CSF leukocytes/mm^3^ | < 1,000 | 1,000-4,000 | ≥ 4,000 |
| CSF neutrophil % | <60 | ≥ 60 |  |
| Blood leukocytes/mm^3^ | < 15,000 | ≥ 15,000 |  |

**Spanos 1989 rule [2]:** Used a fractional polynomial model to determine the probability of ABM (pABM) with cut-off value ≥10%.

pABM = 1/(1+e ^-L^)

where L = 0.52 × number of months from August 1 - 12.76 × CSF-blood glucose ratio (if ratio > 0.6 use 0.6) + 0.341 × (CSF polymorphonuclear neutrophil × 10^6^/L) ^0.333^

+ 2.29 × age + 2.79 (if age < 1 year),

- 2.71 × age + 7.79 (if 1 year < age < 2 years),

- 0.159 × age + 2.69 (if 2 years < age < 22 years) or

+ 0.100 × age - 3.01 (if age > 22 years).

If the Gram stain was positive, a probability of 0.99 was assumed.

**Hoen 1995 or Jaeger 2000 rule [3]:** The rule developed a logistic multivariate model to determine the probability of ABM (pABM) with cut-off value ≥10%.

pABM = 1/(1+e ^-L^), where L=32.13×10^-4^ ×CSF neutrophil count (10^6^/l) + 2.365 ×CSF protein (g/l) + 0.6143 × blood glucose (mmol/l) + 0.2086 × blood leukocyte count (10^9^/l) - 11.

**Freedman 2001 rule [4]:** List of items with cut-off value at one item.

1. Patient’s age (< 6 months)
2. CSF leukocyte count (>30/μl)
3. Peripheral polymorphonuclear count (**>**10×10^3^/μL)
4. CSF glucose concentration (< 40 mg/dL)
5. CSF/serum glucose ratio (< 40%)
6. CSF protein concentration (**>** 45 mg/dL)
7. Positive CSF Gram staining

**Nigrovic 2002 rule [5,6]:** List of items with cut-off value at one item.

1. Positive CSF Gram stain
2. Seizure at or before presentation
3. CSF protein level ≥ 800 mg/L
4. Peripheral absolute neutrophil count ≥ 10,000/μL
5. CSF absolute neutrophil count ≥ 1,000/μL

**Oostenbrink 2004 rule [7]: used a point system with cut-off value ≥8.5 point**

| ***Signs*** | ***Points*** |
| --- | --- |
| Duration of main problem in patient history | 1.0 per day (maximum 10) |
| History of vomiting | 2.0 |
| Cyanosis | 6.5 |
| Disturbed consciousness | 8.0 |
| Meningeal irritation* | 7.5 |
| Petechiae | 4.0 |
| Serum C-reactive protein level, mg per dL (mg per L) |  |
| < 5.0 (50) | 0 |
| 5.0 to 9.9 (50 to 99) | 0.5 |
| 10.0 to 14.9 (100 to 149) | 1.0 |
| 15.0 to 19.9 (150 to 199) | 1.5 |
| ≥ 20.0 (200) | 2.0 |
| **Total:** |  |
|  | |

**Bonsu 2004 rule [8]** developed a fractional polynomial equation to determine the probability of ABM (pABM) with cut-off value ≥10%.

pABM = 1/(1+e ^-L^)

where L = 11.448 + 0.003×CSF neutrophil count (/mm3) - 34.802×(10 ^-2^ ×CSF protein (mg/dL))^0.5^ + 21.991 ×(10 ^-2^ ×CSF protein (mg/dL)) - 0.345 ×age (years).

**Brivet 2005 rule [9]:** List of items with cut-off value at one item.

1. Altered consciousness
2. Seizures
3. Focal neurological findings
4. Shock.

**Schmidt 2006 rule [10]** List of items with cut-off value at two items.

1. CSF leukocyte count ≥ 1000/μL
2. CSF lactate concentration ≥ 3.0 mmol/L
3. CSF protein concentration ≥ 1000 mg/L

**Cauwer 2007 rule [11]:** List of items with cut-off value at one item.

1. CSF neutrophil (%) >80%
2. CSF glucose < 53mg%
3. Blood CRP ≥ 2.0mg%
4. CSF protein ≥ 100mg%.

**Chavanet 2007 rule [12]**: used a point system for children

1. CSF leucocytes count ≥ 1800
2. CSF neutrophil percentage >80
3. CSF protein >1.2 g/L,
4. Glucose CSF/blood ratio ≤ 0.3/μL

| ***Variables*** | ***Points*** |
| --- | --- |
| CSF leucocytes count ≥ 1800 | 2 |
| CSF neutrophil percentage >80 | 3 |
| CSF protein >1.2 g/L | 3 |
| Glucose CSF/blood ratio ≤ 0.3/μL | 3 |

**Lussiana 2011 rule [13]**

Bacterial meningitis is predicted if CSF protein concentration is higher than 50 mg/dl. In case of CSF protein concentration < 0.5g/L, CSF WBC count > 10/µl and/or if CSF glucose concentration is < 40 mg%, there is a strong suspect of bacterial meningitis.

**Tokuda 2009 rule [14]:** Tree decision rule as follow:

***PositiveCSF Gram stain***

**YES**

**NO**

**Low risk of ABM**

***CSF neutrophil***

***Count >150/μL***

**YES**

**NO**

**High risk of ABM**

***Change of mental status***

**YES**

**NO**

**High risk of ABM**

**Low risk of ABM**

**YES**

**High risk of ABM**

**NO**

***CSF neutrophil %***

***>15%***

**References**

1. Thome J, Bovier-Lapierre M, Vercherat M, Boyer P (1980) [Bacterial or viral meningitis? Study of a numerical score permitting an early etiologic orientation in meningitis difficult to diagnose]. Pediatrie 35: 225-236.

2. Spanos A, Harrell FE, Jr., Durack DT (1989) Differential diagnosis of acute meningitis. An analysis of the predictive value of initial observations. Jama 262: 2700-2707.

3. Jaeger F, Leroy J, Duchene F, Baty V, Baillet S, et al. (2000) Validation of a diagnosis model for differentiating bacterial from viral meningitis in infants and children under 3.5 years of age. Eur J Clin Microbiol Infect Dis 19: 418-421.

4. Freedman SB, Marrocco A, Pirie J, Dick PT (2001) Predictors of bacterial meningitis in the era after Haemophilus influenzae. Arch Pediatr Adolesc Med 155: 1301-1306.

5. Nigrovic LE, Kuppermann N, Macias CG, Cannavino CR, Moro-Sutherland DM, et al. (2007) Clinical prediction rule for identifying children with cerebrospinal fluid pleocytosis at very low risk of bacterial meningitis. Jama 297: 52-60.

6. Nigrovic LE, Kuppermann N, Malley R (2002) Development and validation of a multivariable predictive model to distinguish bacterial from aseptic meningitis in children in the post-Haemophilus influenzae era. Pediatrics 110: 712-719.

7. Oostenbrink R, Moons KG, Derksen-Lubsen AG, Grobbee DE, Moll HA (2004) A diagnostic decision rule for management of children with meningeal signs. Eur J Epidemiol 19: 109-116.

8. Bonsu BK, Harper MB (2004) Differentiating acute bacterial meningitis from acute viral meningitis among children with cerebrospinal fluid pleocytosis: a multivariable regression model. Pediatr Infect Dis J 23: 511-517.

9. Brivet FG, Ducuing S, Jacobs F, Chary I, Pompier R, et al. (2005) Accuracy of clinical presentation for differentiating bacterial from viral meningitis in adults: a multivariate approach. Intensive Care Med 31: 1654-1660.

10. Schmidt H, Heimann B, Djukic M, Mazurek C, Fels C, et al. (2006) Neuropsychological sequelae of bacterial and viral meningitis. Brain 129: 333-345.

11. De Cauwer HG, Eykens L, Hellinckx J, Mortelmans LJ (2007) Differential diagnosis between viral and bacterial meningitis in children. Eur J Emerg Med 14: 343-347.

12. Chavanet P, Schaller C, Levy C, Flores-Cordero J, Arens M, et al. (2007) Performance of a predictive rule to distinguish bacterial and viral meningitis. J Infect 54: 328-336.

13. Lussiana C, Loa Clemente SV, Pulido Tarquino IA, Paulo I (2011) Predictors of bacterial meningitis in resource-limited contexts: an Angolan case. PLoS One 6: e25706.

14. Tokuda Y, Koizumi M, Stein GH, Birrer RB (2009) Identifying low-risk patients for bacterial meningitis in adult patients with acute meningitis. Intern Med 48: 537-543.
