# Supplementary material for: Performance of Thirteen Clinical Rules to Distinguish Bacterial and Presumed Viral Meningitis in Vietnamese Children
Source: PLoS One. 2012 Nov 28;7(11):e50341. doi: 10.1371/journal.pone.0050341 (PMC3508924; doi:10.1371/journal.pone.0050341)
Supplement: Table S1 — Theoretical sensitivities and specificities of simple list of items rule calculated and compared using our data set. (DOC) [file pone.0050341.s004.doc]

**Table S1** **Theoretical sensitivities and specificities of simple list of items rule calculated and compared using our data set.**

| Rule | Cut-off value | Observed values | | Theoretical calculation | |
| --- | --- | --- | --- | --- | --- |
|  | Item | Sensitivity % | Specificity % | Sensitivity % | Specificity % |
| Freedman | ≥1 | 98.8 | 14.0 | 99.99 | 10.05 |
| Nigrovic | ≥1 | 96.3 | 56.0 | 96.64 | 53.35 |
| Brivet | ≥1 | 81.3 | 70.0 | 87.46 | 64.58 |
| Schmidt | ≥1 | 85.0 | 82.0 | 93.30 | 81.19 |
| Cauwer | ≥1 | 98.8 | 40.8 | 99.67 | 44.23 |
| Chavanet | ≥1 | 77.5 | 96.0 | 87.46 | 96.00 |
| Normal distribution  (Kolmogorov-Smirnov test) | | p = 0.571 | p = 0.806 | p = 0.981 | p = 0.938 |
| Variance comparison (F-test) | |  |  | p = 0.243^a^ | p = 0.733^b^ |
| Mean comparison  (Paired t-test) | |  |  | p = 0.052^a^ | p = 0.145^b^ |
| ^a^Comparison of sensitivity between data set and theoretical calculation  ^b^Comparison of specificity between data set and theoretical calculation | | | | | |
